# Unmasking Uremic Encephalopathy: Choreoathetoid Movements Mimicking Alcohol Withdrawal in a Person with an Alcohol Use Disorder

**DOI:** 10.7759/cureus.47387

**Published:** 2023-10-20

**Authors:** Keyur Saboo, Rinkle Gemnani, Sourya Acharya, Sunil Kumar, Tushar Sontakke

**Affiliations:** 1 Department of Medicine, Jawaharlal Nehru Medical College, Datta Meghe Institute of Higher Education and Research (Deemed to be University), Wardha, IND

**Keywords:** alcohol withdrawal, case report, movement disorder, encephalopathy, chronic kidney disease (ckd)

## Abstract

Chorea is a disorder characterized by irregular, involuntary movements affecting the limbs, trunk, neck, or face. It can be a significant symptom in various neurologic diseases, including metabolic, autoimmune, and neurodegenerative conditions. The neural foundation that underlies the genesis of chorea appears to be fairly diverse, even though its pathophysiology is frequently associated with the malfunctioning of inhibitory circuits within the basal ganglia. Movement disorders such as tremors, myoclonus, ataxia, chorea, and Parkinsonism may arise due to renal dysfunction or complications from management like renal transplant and hemodialysis. Uremic encephalopathy is a rare but potentially life-threatening neurological complication of chronic kidney disease.

We present a case of a 50-year-old male with a known history of chronic kidney disease and chronic alcoholism, who exhibited abnormal movements resembling chorea upon presentation. Initially suspected as alcohol withdrawal-related chorea, further evaluation revealed concurrent rising creatinine levels, acidosis, and hyperkalemia. Hemodialysis was initiated, resulting in a significant improvement in choreoathetoid movements. This case implies the importance of considering uremic encephalopathy in the differential diagnosis of movement disorders in patients with underlying kidney dysfunction, even in the context of chronic alcoholism.

## Introduction

Prolonged alcohol consumption can lead to movement disorders, including tremors, transient Parkinsonism, choreoathetosis, myoclonus, and dystonia. Tremors are the most commonly known disorder associated with alcohol withdrawal. During the withdrawal process, dyskinesias mainly affect the orofacial muscles and trunk, but there have been choreiform dyskinesias affecting all four limbs. Differential diagnosis of movement disorders in patients with a history of alcoholism can be challenging, often involving conditions such as alcohol withdrawal-related chorea.

Uremic encephalopathy is a neurological syndrome associated with the accumulation of uremic toxins due to impaired renal function. Uremia is a clinical metabolic condition that occurs as a result of renal dysfunction. Neurological symptoms of the condition include confusion, seizures, and movement disorders. It is uncommon for patients with uremia to experience acute hyperkinetic or hypokinetic extrapyramidal conditions [1].

Chorea is a hyperkinetic movement disorder that involves sudden, purposeless movements that rapidly shift from one part of the body to another. It can occur independently or in conjunction with other movement disorders, such as choreoathetosis, a broad term for dyskinesias. Numerous illnesses, roughly split into inherited and acquired ailments, can induce prominent chorea. Although Huntington's disease is the most common cause of adult-onset chorea, autoimmune illnesses are the second most frequent acquired cause after vascular lesions. We present a case highlighting the diagnostic complexity and successful management of uremic encephalopathy presenting as choreoathetosis in a patient with chronic alcoholism [2].

## Case presentation

A 50-year-old man with a 14-year history of chronic alcoholism and known chronic kidney disease on conservative treatment presented to this hospital's emergency department with a two-day history of unusual involuntary movements of his face and limbs. The patient also had a history of diabetes mellitus type 2; he was on regular injectable insulin but was non-compliant to his medication. He also had two episodes of seizure four hours prior to the hospital admission. He had abstained from alcohol for 48 hours at the time of admission and displayed the classic symptoms and signs of alcohol withdrawal syndrome, including generalized tonic-clonic seizure, sweating, anxiety, nausea, and tachycardia. He also expressed concern about his growing weakness, exhaustion, and malaise. He had abruptly discontinued alcohol consumption, and the movements were initially attributed to alcohol withdrawal-related seizure and chorea. Upon examination, he was found to be disoriented and exhibited choreoathetoid movements involving the upper and lower extremities (Video 1). Horizontal nystagmus was present. Hence, treatment was started for alcohol withdrawal syndrome using injectable lorazepam, vitamin B1, levetiracetam, and other supportive medications. The patient's choreoathetoid movements were distressing and continued to worsen despite the initiation of standard alcohol withdrawal treatment.

**Video 1:** Video of the patient showing choreoathetoid movements.

Laboratory investigations revealed elevated serum creatinine and serum urea levels (rising trend) (Table 1 and Table 2) and persistent hyperkalemia. An arterial blood gas analysis confirmed metabolic acidosis. On urine routine examination, the patient also had proteinuria (++) and glucosuria (++). Given the patient's clinical presentation and laboratory findings, the possibility of uremic encephalopathy was considered. Magnetic resonance imaging (MRI) of the brain demonstrated nonspecific changes, including mild cerebral atrophy. Electroencephalography (EEG) showed generalized slowing and intermittent triphasic waves. Cerebrospinal fluid analysis was unremarkable.

**Table 1:** Investigation profile of the patient at the time of admission.

Laboratory parameter	Patient	Reference values
Hemoglobin	8.4 g/dl	13-17 g/dl
Total leukocyte count	6600 /dl	4000-11000/dl
Platelet count	257000/dl	150000-400000/dl
Serum creatinine	4.6mg/dl	0.5-1.2 mg/dl
Serum urea	47 mg/dl	9-20 mg/dl
Serum potassium	3.6 mmol/L	3.5-5.1 mmol/L
Serum sodium	136 mmol/L	137-145 mmol/L
Albumin	2.5 g/dl	3.5-5.0 g/dl
Aspartate aminotransferase	29 U/L	<50 U/L
Alanine aminotransferase	16 U/L	17-59 U/L
Total bilirubin	1.5 mg/dl	0.2-1.3 mg/dl
Serum ammonia	28 (µmol/L)	9-30 (µmol/L)

**Table 2:** Kidney function test of the patient on day 2, on day 3, and after one session of hemodialysis.

Laboratory parameter	Day 2 of admission	Day 3 of admission	After one session of hemodialysis
Serum creatinine	5.6 mg/dl	8.4 mg/dl	3.8 mg/dl
Serum urea	68 mg/dl	98 mg/dl	32 mg/dl
Serum potassium	5.3 mmol/L	5.7 mmol/L	3.7 mmol/L
Serum sodium	135 mmol/L	136 mmol/L	136 mmol/L

With a suspected diagnosis of uremic encephalopathy, urgent hemodialysis was initiated to address the underlying renal dysfunction and facilitate the removal of accumulated uremic toxins. After a single session of hemodialysis, there was a marked improvement in the patient's neurological manifestations just after six hours of its completion, including a significant reduction in choreoathetoid movements (Video 2). The patient's subsequent clinical course was marked by a gradual improvement in mental status, resolution of choreoathetoid movements, and stabilization of metabolic parameters. He received additional hemodialysis sessions as part of his renal replacement therapy. Collaborative care involving nephrology and neurology teams ensured appropriate management and monitoring.

**Video 2:** Video of the patient after one session of hemodialysis showing no choreoathetoid movements.

## Discussion

Multiple studies have examined how alcohol affects the basal ganglia's dopamine metabolism [1]. There is pharmacological evidence for a potential mechanism explaining why Parkinsonism is frequently seen in alcohol withdrawal. Choreoathetosis seems to be significantly rare but could be explained by various personal responses. Chronic alcohol use would be predicted to increase the number of dopamine receptors since it increases dopamine synthesis and lowers dopamine release in the basal ganglia [2]. This might lead to increased receptor activation throughout withdrawal until receptor counts normalize, which could result in the development of a choreoathetoid movement disorder.

Uremic encephalopathy is an organic brain illness that affects the cerebral cortex and is manifested by seizures, tremors, asterixis, multifocal myoclonus, and an altered mental state that can range from mild disorientation to comatose. Basal ganglia involvement is quite uncommon [1]. In diabetic uremic patients, Bhowmick and Lang described a rare clinical syndrome known as acute movement dysfunction brought on by bilateral basal ganglia lesions [3]. Asian patients reported this the most frequently. The presented case illustrates the diagnostic challenges and clinical considerations involved in differentiating between uremic encephalopathy and alcohol withdrawal syndrome, especially when the presentation appears to mimic the latter. Uremic encephalopathy is a rare neurological complication of advanced renal failure, characterized by a broad spectrum of neuropsychiatric manifestations. In patients with chronic alcoholism, the coexistence of renal impairment and alcohol-related neurotoxicity can complicate the clinical picture, necessitating a thorough evaluation to establish the correct diagnosis [4].

The patient's initial presentation with restlessness, agitation, and involuntary movements raised suspicion of alcohol withdrawal syndrome due to his history of chronic alcoholism. The chorea-like movements and hyperreflexia could easily be mistaken for symptoms of alcohol withdrawal, as both conditions can result in similar neuromuscular hyperactivity. However, the lack of response to standard benzodiazepine treatment and the absence of improvement over time prompted the medical team to consider alternative diagnoses.

The patient's history of chronic kidney disease was the key factor that warranted a broader investigation. Uremic encephalopathy occurs when uremic toxins accumulate in the brain due to impaired renal filtration. The patient's deteriorating renal function, as indicated by elevated serum creatinine and blood urea nitrogen (BUN) levels, provided a crucial clue for considering uremic encephalopathy in the differential diagnosis. Chronic alcoholism can lead to renal impairment through multiple mechanisms, such as direct toxic effects of alcohol on the kidneys and dehydration secondary to alcohol consumption [5].

Timely recognition of uremic encephalopathy is crucial, as delayed diagnosis can lead to irreversible neurological damage. In this case, the initiation of hemodialysis upon confirming advanced renal failure resulted in a gradual improvement of the patient's neurological symptoms. Hemodialysis effectively removes uremic toxins from the bloodstream, leading to clinical improvement. This underscores the importance of addressing the underlying cause of the encephalopathy, rather than relying solely on symptomatic management. This case highlights the importance of considering uremic encephalopathy as a potential cause of movement disorders, even in patients with a history of chronic alcoholism [6]. The initial presentation of chorea in this patient, thought to be alcohol withdrawal-related, underscored the complexity of diagnosing underlying medical conditions. The prompt initiation of hemodialysis resulted in a rapid improvement in neurological symptoms, supporting the diagnosis of uremic encephalopathy [7].

This case report emphasizes the need for healthcare professionals to maintain a high index of suspicion for alternative diagnoses in patients with chronic alcoholism who present with neurological symptoms. The overlap between alcohol-related neurotoxicity and uremic encephalopathy highlights the complexity of diagnosing patients with multiple medical comorbidities. A comprehensive evaluation, including a detailed medical history, laboratory investigations, and appropriate imaging studies, is essential for accurate diagnosis and tailored management [8].

## Conclusions

Uremic encephalopathy can present with a wide spectrum of neurological symptoms, often mimicking other neurological disorders. The case of uremic encephalopathy masquerading as alcohol withdrawal in a patient with chronic alcoholism emphasizes the need for a comprehensive diagnostic approach. The clinical presentation of neurological symptoms in the context of chronic alcoholism should prompt clinicians to consider a wide range of differential diagnoses, particularly when the response to initial treatment is inadequate. Swift recognition of atypical presentations, combined with appropriate laboratory investigations and interventions such as hemodialysis, can lead to improved outcomes for patients with complex medical histories and overlapping conditions.
